# Nutritional Evaluation of Cassava (*Manihot esculenta*) Peels as a Dietary Supplement in Tropical* Friesian Holstein* Cross Breed Dairy Cattle

**DOI:** 10.1155/2019/6517839

**Published:** 2019-04-09

**Authors:** Herinda Pertiwi, Nisha Yulanda Maharsedyo, Lestiana Amaro, Tri Bhawono Dadi

**Affiliations:** ^1^Veterinary Paramedic Division, Health Departement, Faculty of Voccational Studies, Airlangga University, Surabaya, Indonesia; ^2^Veterinary Clinic Department, Faculty of Veterinary Medicine, Airlangga University, Surabaya, Indonesia

## Abstract

Cassava (*Manihot esculenta*) Peels supplementation in feed was evaluated on quality and quantity of dairy cattle production. Cassava peels were waste product of tapioca flour industry. A total of 26 lactation dairy cattle breed Friesian Holstein cross breed maintained at individual cage at farm on Kawi Mountain, Malang, East Java. It was randomly allotted to treatment and control group (13 head of cattle in each group). Treatment group were fed with cassava peels base dietary, whereas control group were fed by grass and commercial ration dietary. After a month, the quality and quantity of milk were recorded daily for a month and analyzed on automatic milk analyzer. The result showed significant changes (*p<0.05*) on treatment group were shown on percentage of protein (2.87%), lactose (4.40%), solid nonfat (8.49%), and total solid (12.23%). This quality of milk only needed cost of 1.15 €/head/day compared with control group which needed 2.02 €/head/day. These results indicated that cassava peels in feed can produce higher total solid and protein level of milk than control group. They also have high potential as a feed source to cut production cost; therefore, the farmer income increased slightly.

## 1. Introduction

Maintenance of dairy cows in tropical climates with high temperatures and humidity can reduce livestock productivity and milk production [1]. Nutritional requirement of lactating dairy cows is closely related to body weight and milk production [2].

Feed intake is one of the main factors that influence milk production. Dairy cows have to maximize the amount of feed intake during lactation, so that the milk productivity will increase both quality and quantity. The problem is how to increase the productivity of dairy cattle to produce high quality dairy products; this is also supported by feed intake that has high nutritional value and quality, but usually it is very expensive. Based on these problems it is necessary to provide cheap feed by prioritizing the nutritional value and quality of the dairy cattle.

Cassava (*Manihot esculenta*) has a good potential to be used as a high protein supplement feed at a relatively cheap price. Cassava plants that have been dried, especially the leaves and tubers, are high sources of protein and can be used as a supplement to ruminant nutrition, especially in dairy cows, beef cattle, and buffalo [3, 4]. The administration can be directly as a feed supplement and as a source of protein in concentrates [5, 6]. By-pass protein of cassava in the rumen can an increase in milk fat and protein content [3]. However study about cassava peel supplementation in feed have limited data, but in Indonesia there are some tapioca flour industries which have cassava peel as their waste product. Therefore, the authors were interested to do a research about cassava peel supplementation to the milk quality and quantity of dairy cattle in tropical climate.

## 2. Materials and Methods

Twenty-six cross breed Friesian Holstein (FH) dairy cattle averaging 130 DIM were used. Animals were divided into two groups assigned to 2 treatments randomly within groups to evaluate the response to cassava peels supplementation. Cows are housed in a tie-stall barn, fed, and milked at 05.30 am and 15.00 pm. All animals had free access to drinking water. The experiment lasted 50 days, 20 days for adaptation, 30 days for data collection. The nutrients, ingredients, and composition of experimental diets are presented in Table 1. All samples consumed 39–44,5 kg ration and grass. P1 were fed by complete feed containing 34,5% cassava peels, and P2 were fed without cassava peels.

Milk weight was recorded at each milking on the morning and afternoon. A 50 ml aliquots of milk were collected daily as each milking proportionally to yield. It is analyzed for fat, protein, lactose, total solid, and solid nonfat using automatic milk analyzer. Feed cost and overfeed income cost were also calculated. The means were compared with independent t-Test for significant differences among treatments using SPSS software 16.0.

## 3. Results and Discussions

Table 2 indicated that cassava peels dietary in ration could change significantly (p<0.05) on protein, lactose, solid nonfat (SNF), and total solid (TS). It did not give significant differences (p>0.05) on fat, nAmount, milk volume, daily feed cost, and IOFC. However, Indonesia national minimum standard for fresh milk are fat 3%, SNF 8%, protein 2.7%, and TS 11% [7]. Unchanged milk volume on the supplementation of cassava peels, cassava leaves, and cassava roots was also reported in the studies of Petlum et al. [8], Lunsin et al. [9], and Ukanwoko and Ibeawuchi [10]

Milk components are synthesized in alveoli cells from simple substrates derived from crude fiber of feed. High crude fiber in feed will produce high amounts of acetic acid in the rumen [11]. When the production of acetic acid in the rumen decreases, it will bring low levels of milk fat. The fat content of milk by crude fiber fibers in the ration, if the level of low crude fiber will produce the level of fat produced, because crude fiber contains neutral detergent fiber (NDF) which is raw material of acetic acid produced in the rumen; then it synthesized to be milk fat [12]. Very high NDF content can produce high levels of milk fat, because the crude fiber in the rumen will be degraded by rumen microbes; therefore, it produce more acetic acid than propionic acid [13].

Cassava leaves dietary as much as 1.5 kg/head/day increase in milk quality significatly [14], because the cassava leaves were source of dissolved carbohydrates and by-pass protein. Carbohydrate is a precursor of glucose which is used by rumen microorganisms to produce VFA (Volatile Fatty Acid) including acetic acid to form milk fat. Crude fiber content of dairy cattle ration should not be less than 13% because it can reduce the fat content of milk produced [15].

The high level of milk protein in of P1 group is in line with the amount of cassava peels given, because in cassava peels protein (33.3%) and amino acids that are easily degraded by rumen microbes are valine (5.6%), leucine (8.3%), and isoleucine (4,2%). Cassava peels are rich in soluble protein (Jalaudin, 1994). Milk protein increase level could be caused by condensed tannin content in cassava which plays a role to form complex tannins and increases by-pass protein in the rumen [16].

The ability of tannins to precipitate proteins due to tannins has a number of functional group groups that can form strong complexes with protein molecules. The complex bonds are difficult to ferment by the rumen microbes by inhibiting enzymes that degrade cell walls. Tannins can increase the value of the benefits of protein source feed by ruminants, through reduced degradation of protein in the rumen [17].

Solid nonfat (SNF) levels of group P1 (8.49%) were higher than P2 (8.46%); SNF is influenced by levels of lactose and protein [18]. If lactose and milk protein levels are high, SNF will increase. Milk protein is produced by feed consumed synthesized by rumen microbes into amino acids and these amino acids are absorbed in the small intestine and channeled into the blood and into udder secretion cells becoming milk protein [19]. Addition of protein source in feed can increase levels of SNF, because the protein milk content also increases [19, 20].

We have high volatile fatty acid (VFA) degradation along with increasing of NH3 production so that NH3 can be used for rumen microbial protein synthesis [21]. Most carbohydrates will be fermented into VFA by cellulotic microbes in the rumen [22], VFA containing acetic acid, propionate acid, and butyrate acid. Volatile fatty acid is a by-product of microbial activity in rumen [23]; it is associated with livestock productivity because most of the VFA in the rumen comes from feed carbohydrate fermentation [24]. VFA is the result of crude fiber fermentation of feed which source of energy for ruminants and as a carbon framework for microbial growth in the rumen. Wanapat [25] reported that cassava top silage dietary significantly affected ruminal fermentation end-products, especially increased propionate production, decreased protozoal population, and suppressed methane production.

Supplementation of cassava peels in dairy cattle feed was better than the use of tofu dregs, tofu dregs containing proteins that are difficult to degrade in the rumen at a rate of degradation of 9.8% per hour, and average production speed of N. The net ammonia is 0.667 mM per hour [26], because it has undergone coagulation and denaturation due to heating during the tofu making process; therefore, the tofu dregs protein is difficult to convert into ammonia. This causes the growth of rumen microbes to be obstructed. In addition, cassava contains a natural content of lactic acid bacteria and yeast, a minimum mycotoxin contamination and a low, nontoxic content of hydrocyanide (HCN) which promotes good health in animals [27–29]; it also increase in lactoperoxidase content and reduction in coliform bacteria in the milk [27, 30–33].

As much as 87-89% of the milk composition, total solid (TS) level is only 11-13%; if TS is high, other nutrients of milk such as lactose, protein, minerals, and vitamins will be also high [34]. Total solid group P1(12.0%) lower than P2 (12.24%); it was because P1 have SNF 3.51% and fat 8.49%. Solid nonfat contains protein, lactose, mineral, and vitamin. Total solid value was obtained from SNF plus fat level. In Indonesia, level of TS will determine the price of milk. Higher TS will give farmer more income, so that farmer always does anything to increase milk TS level of their dairy cattle.

## 4. Conclusion

The results indicated that cassava peels in feed can produce higher total solid and protein level of milk than control group. It also has high potential as a feed source to cut production cost; therefore, the farmer income increased slightly.

## Figures and Tables

**Table 1:** Ingredients and chemical composition of the treatments ration.

Materials	P1 (%)	P2 (%)
Odot grass (*Pennisetum purpureum*)	34.5	57.4
Cassava peel	34.5	0
Cassava	12.9	0
Berr waste product	17.59	0
Salt	0.33	0
Corn straw silage	0	12.6
Complete feed fermentation	0	19.3
Commercial ration	0	10.7
*Nutrition*		
Dry Matter	37.7	66.07
Crude protein	10.61	11.50
Crude fat	3.97	8
Crude fiber	14.25	19.81
Total digestible nutrient	65.90	58.48

**Table 2:** Milk quality and quantity of the treatments group.

Parameters	P1	P2
Fat (%)	3.51	3.78
Protein (%) S	2.87^a^	2.86^b^
Lactose (%) S	4.43^a^	4.48^b^
Solid nonfat (%) S	8.49^a^	8.46^b^
Total Solid (%) S	12.0^a^	12.24^b^
Volume / head (L) NS	11.35^a^	13.15^a^
Feed cost/head/day (€) NS	1.15^a^	2.02^a^
Income Over Feed Cost (€) NS	2.11^a^	1.72^a^

Mean values bearing the same superscript in columns did not differ significantly; NS, not significant (P>0.05) and S, significant.

## Data Availability

(1) The main data of milk productivity in this research was measured in the dairy cattle farmer located in the Kawi Mountain Malang Indonesia. The farmer has agreed to do this research with our team; he facilitated and gave full access to prepare, do, and evaluate this study in his farm. (2) The main data of milk quality such us milk fat, protein, lactose, solid not fat, and total solid was analyzed by automatic milk analyzer in the quality control laboratorium of PT. Greenfield Indonesia integrated with dairy cattle farmers in the Kawi Mountain as partnership of community services responsibility of industry. (3) Secondary data of ration formula and its nutrient was measured in the faculty of veterinary medicine, Airlangga University, Indonesia. It was important to explore the effect of cassava peels supplementation on the feed nutrient which is given to the cattle. (4) Supportive data to discuss and compare the main data was from previous studies and dataset, which have been cited from recent journal related to this article focus. These data are publicly available and accessible online. Detailed source has been written on reference on the manuscript. (5) Detailed fatty acids and amino acid data used to support the finding of this study have not been made available because the technology to analyze it was expensive in Indonesia; we did not have enough funding to do this analysis.
